# Interleukin-16 is increased in dialysis patients but is not a cardiovascular risk factor

**DOI:** 10.1038/s41598-024-61808-7

**Published:** 2024-05-17

**Authors:** Frederic Brösecke, Anja Pfau, Theresa Ermer, Ana Beatriz Dein Terra Mota Ribeiro, Lisa Rubenbauer, Veena S. Rao, Sarah Burlein, Bernd Genser, Martin Reichel, Peter S. Aronson, Steven Coca, Felix Knauf

**Affiliations:** 1 Department of Nephrology and Medical Intensive Care, Corporate Member of Freie Universität Berlin and Humboldt-Universität zu Berlin, Charité – Universitätsmedizin Berlin, Campus Charité Mitte, Charitéplatz 1, 10117 Berlin, Germany; 2 MVZ Dialysezentrum (Dialysis Center), Schweinfurt, Germany; 3 Department of Nephrology and Hypertension, Friedrich-Alexander-Universität Erlangen-Nürnberg, Erlangen, Germany; 4 Department of Internal Medicine, Section of Nephrology, Yale University School of Medicine, New Haven, CT USA; 5 Department of Internal Medicine, Section of Cardiovascular Medicine, Yale University School of Medicine, New Haven, CT USA; 6 Department of General Medicine, Centre for Preventive Medicine and Digital Health Baden Württemberg, Ruprecht Karls University, Heidelberg, Germany; 7 High5Data GmbH, Heidelberg, Germany; 8 Mount Sinai School of Medicine, Mt. Sinai Hospital, New York, NY USA

**Keywords:** Chronic kidney disease, Dialysis, Interleukin-16, Cytokines oxalate, Cardiovascular events, Haemodialysis, Kidney diseases, Chronic kidney disease, Chronic inflammation

## Abstract

Oxalate, a uremic toxin that accumulates in dialysis patients, is associated with cardiovascular disease. As oxalate crystals can activate immune cells, we tested the hypothesis that plasma oxalate would be associated with cytokine concentrations and cardiovascular outcomes in dialysis patients. In a cohort of 104 US patients with kidney failure requiring dialysis (cohort 1), we measured 21 inflammatory markers. As IL-16 was the only cytokine to correlate with oxalate, we focused further investigations on IL-16. We searched for associations between concentrations of IL-16 and mortality and cardiovascular events in the 4D cohort (1255 patients, cohort 2) and assessed further associations of IL-16 with other uremic toxins in this cohort. IL-16 levels were positively correlated with pOx concentrations (*ρ* = 0.39 in cohort 1, r = 0.35 in cohort 2) and were elevated in dialysis patients when compared to healthy individuals. No significant association could be found between IL-16 levels and cardiovascular events or mortality in the 4D cohort. We conclude that the cytokine IL-16 correlates with plasma oxalate concentrations and is substantially increased in patients with kidney failure on dialysis. However, no association could be detected between IL-16 concentrations and cardiovascular disease in the 4D cohort.

## Introduction

The risk of morbidity and mortality in patients with kidney failure requiring maintenance dialysis is extremely high as compared to the general population^1,2^. Chronic inflammatory processes in these patients inordinately increase cardiovascular risk^3,4^. Previous studies have demonstrated an alteration of the cytokine network, with tumour necrosis factor alpha (TNF-α), interleukin (IL)-1, IL-6 and IL-8 being the cytokines for which an increase in dialysis patients has been most consistently described in the literature^3,5–7^. However, underlying triggers and mechanisms of cytokine release are not completely understood.

Oxalate is a uremic toxin that is mainly excreted via the kidney and accumulates in patients with declining glomerular filtration rate^8^. In patients on maintenance dialysis, its concentration can reach levels that are almost tenfold higher compared to healthy individuals^9,10^. Clinical and experimental evidence suggests that oxalate promotes the progression of chronic kidney disease^11^, atherosclerosis^12^ and cardiovascular complications^13,14^. Calcium oxalate crystals were also identified as triggers of inflammation and increased IL-1β secretion^15,16^. In addition, IL-1α is released in response to oxalate crystals^17^ and has been suggested to play a central role in cardiovascular disease^17^. These data suggest that oxalate might provide a link between uremia, inflammation and subsequent clinical complications including cardiovascular disease. The present work aims to determine the oxalate-associated cytokine pattern in patients with kidney failure requiring chronic dialysis and to investigate its potential impact on cardiovascular disease.

## Results

### Out of 21 cytokines, IL-16 is substantially increased and correlates with plasma oxalate (pOx) concentration

In cohort 1, pOx concentrations and cytokine levels were obtained from 104 dialysis patients; the baseline characteristics of the cohort are described elsewhere^14^: in brief, patients had a median pOx concentration of 23.76 µM (interquartile range (IQR) 10.0), were on average 65.9 (standard deviation (SD) 14.6) years old and were on dialysis for a median time of 41.5 months (IQR 51.8). Almost 78% received hemodialysis (HD) three times a week, 22% were on peritoneal dialysis (PD)^14^.

**Figure 1:**
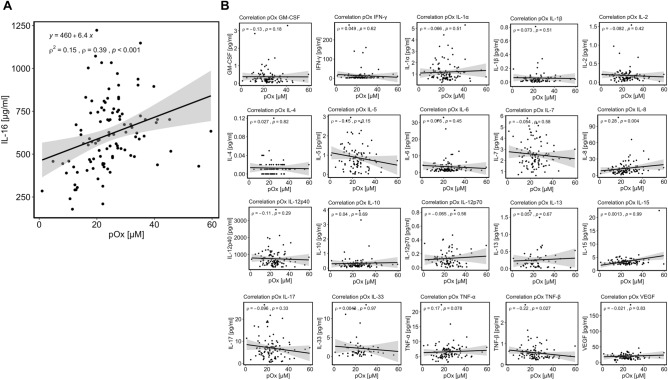
Interleukin-16 correlates with plasma oxalate concentrations in patients with kidney failure requiring long-term dialysis (cohort 1). In 104 patients with kidney failure requiring long-term dialysis in the US (cohort 1), plasma oxalate (pOx) concentration, measured by enzymatic assay, and 21 cytokines, measured by a V-PLEX Proinflammatory Panel 1 Human, a V-PLEX Cytokine Panel 1 Human, and an Interleukin(IL)-33-ELISA, were assessed. (**A**) IL-16 was the only cytokine that correlated with pOx. Scatter plot with regression line (black line). Spearman`s rank test, ρ = 0.39, *p* < 0.001. (**B**) The levels of the remaining 20 cytokines did not significantly correlate with pOx concentration. Scatter plots with regression lines and results of the Spearman correlation analysis (Spearman`s rank test; ρ and *p*-value).

To examine the cytokine profile associated with pOx concentrations, we conducted an analysis using a panel of diverse cytokines, including pro-inflammatory, anti-inflammatory, and chemotactic mediators. Out of 21 cytokines examined, IL-16 was the only one that correlated with pOx (Fig. 1A, *ρ* = 0.39, *p* < 0.001 and Table 1).

**Table 1:** Correlation of pOx and a panel of 21 cytokines in a cohort of 104 US patients on dialysis (Cohort 1).

	Spearman correlation		Linear regression					
			Model 1 (unadjusted)			Model 2 (adjusted for age, gender, BMI, Diabetes.)		
	Rho estimates	*P* value	β coefficient (SE)	95% CI	*P* Value	β coefficient (SE)	95% CI	*P* Value
GM-CSF	− 0.13	0.18	0 (0)	− 0.01; 0.01	0.96	0 (0)	− 0.01; 0.01	0.96
IFN-γ	0.05	0.62	− 0.24 (0.37)	− 0.96; 0.49	0.52	− 0.2 (0.38)	− 0.94; 0.55	0.60
IL-1α	− 0.07	0.51	0 (0.01)	− 0.01: 0.02	0.66	0 (0.01)	− 0.01; 0.02	0.67
IL-1β	0.07	0.51	0 (0)	0; 0	0.84	0 (0)	0; 0	0.80
IL-2	− 0.08	0.42	0 (0)	− 0.01; 0	0.58	0 (0)	0; 0	0.66
IL-4	0.03	0.82	0 (0)	0; 0	0.85	0, 0	0; 0	0.95
IL-5	− 0.15	0.15	− 0.01 (0.01)	− 0.02; 0	0.12	− 0.01 (0.01)	− 0.02; 0	0.14
IL-6	0.08	0.45	− 0.03 (0.05)	− 0.13; 0.07	0.57	− 0.02 (0.05)	− 0.12; 0.08	0.69
IL-7	− 0.05	0.59	− 0.01 (0.01)	− 0.03; 0.01	0.36	− 0.01 (0.01)	− 0.03; 0.01	0.42
**IL-8**	**0.28**	**0.004**	0.2 (0.13)	− 0.07; 0.46	0.15	0.2 (0.14)	− 0.08; 0.48	0.17
IL-10	0.04	0.69	0 (0.01)	− 0.01; 0.01	0.79	0 (0.01)	− 0.01; 0.01	0.76
IL-12p40	− 0.11	0.29	− 2 (4.85)	− 11.62; 7.63	0.68	− 1.65 (4.92)	− 11.42; 8.13	0.74
IL-12p70	− 0.07	0.56	0 (0)	0; 0	0.57	0 (0)	0; 0	0.55
IL-13	0.06	0.67	0 (0)	− 0.01; 0.01	0.70	0 (0)	− 0.01; 0.01	0.73
IL-15	0.00	0.99	0.06 (0.02)	0.02; 0.1	0.003	0.06 (0.02)	0.02; 0.11	**0.003**
**IL-16**	**0.39**	** < 0.001**	**6.39 (2.03)**	**2.37; 10.42**	**0.002**	**6.72 (1.99)**	**2.77; 10.67**	**0.001**
IL-17	− 0.10	0.33	− 0.07 (0.05)	− 0.16; 0.03	0.18	− 0.07 (0.05)	− 0.17; 0.02	0.14
IL-33	0.00	0.98	− 0.02 (0.04)	− 0.1; 0.05	0.54	− 0.03 (0.04)	− 0.12; 0.05	0.45
TNF-α	0.17	0.08	0.01 (0.02)	− 0.04; 0.06	0.60	0.01 (0.03)	− 0.04; 0.06	0.67
TNF-β	− 0.22	0.03	− 0.01 (0)	− 0.01; 0	0.12	− 0.01 (0)	− 0.01; 0	0.15
VEGF	− 0.02	0.83	0.09 (0.18)	− 0.27; 0.45	0.62	0.1 (0.19)	− 0.28; 0.47	0.61

pOx: plasma oxalate; GM-CSF: granulocyte-macrophage colony-stimulating factor; IFN: interferon; IL: interleukin; TNF: tumour necrosis factor; VEGF: vascular endothelial growth factor; BMI: body mass index; SE: standard error, CI: confidence interval, *p*-value below 0.01 considered statistically significant; Model 1: unadjusted, Model 2: adjusted for gender, age, BMI, Diabetes mellitus.

In multiple linear regression models, IL-16 was strongly associated with pOx after adjusting for the potential main confounders such as age, gender, body mass index (BMI), and diabetes (model 2, β = 6.72, 95% CI 2.77–10.67). The correlation analysis of the remaining 20 cytokines can be seen in Fig. 1B.

Table 2 presents the characteristics of the US cohort stratified by tertials of IL-16 concentrations.

**Table 2:** Characteristics of 104 US patients with kidney failure requiring chronic dialysis (Cohort 1) stratified by IL-16 tertiles.

	First tertile ≤ 518.29 pg/mL n = 35	Second tertile 518.3–700.8 pg/mL n = 34	Third tertile ≥ 700.81 pg/mL n = 35	*P* value
IL-16 [pg/mL]	398.6 (87)	603.7 (54)	841.2 (137)	** < 0.001**
pOx [µM]*	18.9 (11)	24.4 (10)	25.9 (7)	**0.003**
Age [years]	67.9 (14)	66.9 (14)	63 (16)	0.163
Male, No. (%)	19 (18)	23 (22)	11 (11)	0.01
Ethnic background, No. (%)				
White	14 (13)	16 (15)	17 (16)	0.625
Black	18 (17)	16 (15)	17 (16)	
Hispanic	2 (2)	2 (2)	0	
Other	1 (1)	0	0	
Dialysis mode, No. (%)				
HD	26 (25)	28 (27)	27 (26)	0.716
PD	9 (8.7)	6 (5.8)	8 (7.7)	
Weight [kg]	88.2 (21)	93.0 (26)	86.4 (29)	0.757
BMI [kg/m^2^]	31.6 (8.0)	31.2 (7.6)	31.4 (9.4)	0.926
Diabetes, No. (%)	22 (65)	22 (69)	23 (66)	0.937
Time on dialysis [months]*	27 (32)	42 (54)	48 (49)	**0.003**
Urine output, No. (%)				
Diuresis > 300 mL/day	18 (17)	7 (7)	3 (3)	** < 0.001**
Diuresis < 300 mL/day	14 (13)	23 (22)	28 (27)	
Not defined	3 (3)	4 (4)	4 (4)	
WBC count [1000/µl]	7.0 (1.9)	6.5 (2.0)	6.9 (2.4)	0.845
BUN [mg/dl]	50.7 (18)	54.8 (20)	53.6 (15)	0.513
Albumin [g/dl]	3.7 (0.4)	3.9 (0.3)	3.8 (0.4)	0.556
CRP [mg/l]*	3.5 (5.8)	2.8 (17.9)	2.1 (12.7)	0.318
Creatinine [mg/dl]*	7.4 (2.2)	9.6 (4.9)	9.6 (4.7)	0.149
Use of Diuretics, No. (%)	11 (29)	10 (29)	6 (17)	0.213
Use of Phosphate binder, No. (%)	25 (71)	32 (94)	30 (86)	0.062
Use of Antihypertensive drugs, No. (%)	30 (86)	27 (79)	29 (83)	0.904

HD: haemodialysis; PD: peritoneal dialysis; BMI: body mass index, calculated as weight in kilograms divided by height in meters squared; WBC: white blood cells; BUN: blood urea nitrogen; CRP: C-reactive protein.

Continuous variables are expressed as mean (SD) or median (IQR)* where appropriate, categorical variables as No. (%).

Statistical analysis: *p*-value of ANOVA F statistic (for continuous outcomes) or Pearson chi square statistic (for categorical outcomes).

Patients with higher IL-16 levels tended to have a longer dialysis vintage (*ρ* = 0.29, Supplementary Fig. 1A). In addition, they were less likely to have residual kidney function defined as urine output > 300 mL/day (662 pg/mL, SD 201, vs. 482 pg/mL, SD 162, Supplementary Fig. 1B). IL-16 levels of patients treated with HD (619 pg/mL, SD 191) and PD (599 pg/mL, SD 259) were not statistically different.

The analysis of consecutive dialysis sessions showed little variation in the analytes of interest (Supplementary Table 1).

When examining the survival probability over a 2.5-year follow-up period, no significant difference was observed between the upper and lower IL-16 median groups (Supplementary Fig. 2).

### Validation of elevated IL-16 and pOx concentration in a small cohort of dialysis patients

To confirm our findings, we repeated the measurement of pOx and IL-16 in a small cohort of 12 patients on maintenance dialysis in Germany compared with six healthy controls (Supplementary cohort), using a complementary method for detection of IL-16 (IL-16-Quantikine ELISA). All 12 patients received HD thrice weekly and were on average 67.3 years (SD 15.5) old, with the control group being on average 25 years younger (40.2 years, SD 12, Supplementary Table 2).

We were again able to demonstrate a significant correlation between IL-16 and pOx (*ρ* = 0.81, Supplementary Fig. 3A).

Moreover, IL-16 concentrations in HD patients (521.5 pg/mL, SD 184) were substantially elevated when compared to the levels of six healthy individuals (150.1 pg/mL, SD 27), as well as pOx (19.55 µM IQR 29.4 vs. 1.2 µM IQR 0.9) (Supplementary Fig. 3B).

### Higher IL-16 levels do not correlate with a higher risk for cardiovascular events in the 4D cohort

A total of 1102 patients had measurements of IL-16 concentrations. The median (IQR) IL-16 concentration at baseline was 417 (208) pg/mL with no significant difference between the atorvastatin and placebo group. During follow-up, 538 patients died (48.8%). A total of 344 patients reached the primary composite cardiovascular endpoint with myocardial infarction (fatal or non-fatal) and stroke (fatal or non-fatal) occurring in 182 and 86 patients, respectively. 141 patients died from sudden cardiac death, 37 due to congestive heart failure.

The baseline patient characteristics are shown in Table 3.

**Table 3:** Patient characteristics stratified by quartile of IL-16 at baseline; study population n = 1102 (Cohort 3, 4D cohort, n = 1102).

	Quartile 1 ≤ 326 pg/mL, n = 273	Quartile 2 327–416 pg/mL, n = 278	Quartile 3 417–535 pg/mL, n = 275	Quartile 4 536–1377 pg/mL, n = 276	*P* value
IL-16 [pg/mL]	261.0 (51.8)	370.2 (25.4)	471.6 (33.5)	714.3 (170.8)	
Age [years]	67.1 (7.5)	66.6 (9.03)	66.4 (7.9)	65.3 (8.6)	0.096
Sex [Male], No. (%)	53	52	51	57	0.854
Atorvastatin treatment, No. (%)	49	49	51	48	0.777
Systolic blood pressure [mmHg)]	144.0 (20.8)	144.8 (22.4)	146.1 (21.6)	148.2 (22.1)	0.123
Diastolic blood pressure [mmHg]	75.6 (10.2)	75.5 (11.1)	76.3 (10.1)	76.5 (12.3)	0.672
Body mass index [kg/m^2^]	27.5 (4,7)	27.9 (4.9)	27.6 (4.6)	27.2 (4.7)	0.379
Duration of diabetes [years]	17.4 (9.1)	18.0 (8.3)	18.1 (8.4)	18.6 (8.8)	0.805
Time on dialysis [months]	7.1 (6.3)	7.9 (6.7)	8.4 (7.4)	9.7 (7.3)	** < 0.001**
Smoker, No. (%)	5	9	9	10	0.611
Coronary artery disease, No. (%)	34	31	27	26	0.060
Congestive heart failure, No. (%)	37	36	37	33	0.080
Peripheral vascular disease, No. (%)	44	46	43	48	0.483
Cholesterol total [mg/dL]	220.1 (41.0)	218.8 (46.0)	275.0 (41.8)	223.4 (41.0)	0.523
LDL-Colesterol [mg/dL]	124.9 (30.4)	123.4 (30.8)	126.7 (28.9)	129.5 (28.7)	0.097
HDL-Colesterol [mg/dL]	35.5 (12.1)	35.3 (12.2)	37.2 (14.5)	37.0 (14.1)	0.218
Albumin ([g/dL]	3.8 (0.3)	3.8 (0.3)	3.8 (0.3)	3.9 (0.3)	** < 0.001**
CRP* [µg/mL]	4.7 (8.7)	4.9 (8.7)	5.8 (8.6)	11.1 (7.3)	** < 0.001**
Triclycerides [mg/dL]	271.9 (164.1)	271.3 (175.4)	254.8 (168.3)	251.7 (140.6)	0.312
Leukocytes [× 10^3^/µL]	7.4 (2.1)	8.0 (2.4)	8.3 (2.2)	8.7 (2.6)	** < 0.001**
HbA1c [%]	6.7 (1.2)	6.8 (1.3)	6.7 (1.3)	6.8 (1.3)	0.899
ADMA [µmol/L]	0.8 (0.1)	0.9 (0.2)	0.9 (0.2)	0.9 (0.2)	** < 0.001**
Oxalate [µM]	39.5 (22.5)	41.4 (19.6)	49.3 (29.1)	58.7 (27.0)	** < 0.001**
NT-proBNP [pg/mL]	5753 (10,897)	7166 (10,616)	8677 (13,472)	11,114 (16,157)	** < 0.001**
Creatinine [mg/dL]	6.1 (2.1)	6.6 (2.1)	7.0 (2.3)	7.9 (2.2)	** < 0.001**
Use of diuretics, No. (%)	84	87	78	73	** < 0.001**
Use of ACE Inhibitors, No. (%)	54	40	45	46	0.251
Use of antiplatelet treatment, No. (%)	50	53	52	51	0.845

CRP: C-reactive protein. ADMA: Asymmetric dimethylarginine. NT-proBNP: N-terminal pro-B-type natriuretic peptide. ACE: angiotensin-converting enzyme. Continuous variables are expressed as mean (SD) or median (IQR)* where appropriate, categorical variables as No. (%). *P* value was obtained by analysis of variance model for continuous variables and Pearson chi-square test for categorical variables.

Patients with the highest IL16- concentrations at baseline (4th quartile) tended to have higher levels of C-reactive protein (CRP), oxalate, N-terminal pro-B-type natriuretic peptide (NT-proBNP) and asymmetric dimethylarginine (ADMA) (*P* < 0.001) compared to those with the lowest IL-16 levels (1st quartile). They also had longer dialysis vintage, and were less likely to have residual kidney function, as indicated by “use of diuretics” (*P* < 0.001).

Again, we were able to confirm the correlation between oxalate and IL-16 levels (r = 0.35, *p* < 0.001). When examining additional bivariate correlations of IL-16 with other uremic toxins (namely creatinine, ADMA, SDMA, serum carbamylated albumin (C-Alb), and BUN/Urea), we found the strongest correlation with C-Alb (r = 0.31), followed by creatinine (r = 0.28) and SDMA (r = 0.19). ADMA exhibited only a very weak correlation (r = 0.08). A multivariate prediction model for IL-16 revealed a similar ordering of predictive factors. The most important factor was pOx predicting IL-16 with a beta coefficient of 0.25. 71% of the observed bivariate correlation between IL-16 and pOx is not attributable to the uremic toxins creatinine, ADMA, SDMA, and C-Alb.

However, patients with higher IL-16 concentrations did not have a higher risk of mortality or to reach the primary composite outcome of the 4D study, of combined cardiovascular events, as shown in Table 4.

**Table 4:** Risk of all-cause mortality, combined cardiovascular events, death due to heart failure, sudden cardiac death, myocardial infarction, stroke, and death due to infection, stratified by quartiles of IL-16 concentration at baseline (Cohort 3, 4D cohort, n = 1102).

Outcome	HRs stratified by IL-16 quartiles at baseline				
	Quartile 1	Quartile 2	Quartile 3	Quartile 4	Global
	≤ 326 pg/mL	327–416 pg/mL	417–535 pg/mL	536–1377 pg/mL	*P*-value
	n = 273	n = 278	n = 275	n = 276	
All-cause mortality					
Crude HR (95% CI)	1	0.86 (0.67–1.11)	0.83 (0.66–1.05)	0.98 (0.76–1.26)	0.88
Adjusted^a^ HR (95% CI)	1	0.85 (0.67–1.09)	0.82 (0.65–1.04)	1.01 (0.80–1.28)	0.93
Adjusted^b^ HR (95% CI)	1	0.80 (0.61–1.04)	0.78 (0.61–1.00)	0.92 (0.71–1.19)	0.52
Cardiovascular events^c^					
Crude HR (95% CI)	1	0.80 (0.59–1.07)	0.78 (0.59–1.03)	0.93 (0.69–1.25)	0.62
Adjusted^a^ HR (95% CI)	1	0.78 (0.58–1.05)	0.77 (0.59–1.01)	0.93 (0.69–1.25)	0.62
Adjusted^b^ HR (95% CI)	1	0.76 (0.55–1.05)	0.74 (0.55–0.99)	0.83 (0.59–1.16)	0.27
Sudden cardiac death					
Crude HR (95% CI)	1	0.96 (0.60–1.54)	0.87 (0.54–1.40)	1.30 (0.24–0.84)	0.24
Adjusted^a^ HR (95% CI)	1	0.96 (0.59–1.54)	0.86 (0.54–1.38)	1.36 (0.88–2.10)	0.17
Adjusted^b^ HR (95% CI)	1	0.96 (0.59–1.56)	0.90 (0.55–1.46)	1.41 (0.89–2.23)	0.14
Death due to heart failure					
Crude HR (95% CI)	1	0.53 (0.20–1.39)	0.42 (0.15–1.20)	1.26 (0.54–2.93)	0.59
Adjusted^a^ HR (95% CI)	1	0.52 (0.20–1.36)	0.43 (0.15–1.29)	1.37 (0.58–3.20)	0.47
Adjusted^b^ HR (95% CI)	1	0.41 (0.15–1.14)	0.35 (0.12–1.01)	0.94 (0.37–2.39)	0.9
Myocardial infarction					
Crude HR (95% CI)	1	0.90 (0.61–1.32)	0.85 (0.58–1.26)	0.75 (0.50–1.13)	0.17
Adjusted^a^ HR (95% CI)	1	0.89 (0.60–1.30)	0.85 (0.57–1.25)	0.74 (0.48–1.15)	0.18
Adjusted^b^ HR (95% CI)	1	0.85 (0.57–1.26)	0.82 (0.55–1.22)	0.70 (0.44–1.12)	0.14
Stroke					
Crude HR (95% CI)	1	0.66 (0.36–1.23)	0.58 (0.30–1.14)	0.86 (0.52–1.44)	0.57
Adjusted^a^ HR (95% CI)	1	0.66 (0.36–1.22)	0.57 (0.30–1.10)	0.96 (0.58–1.58)	0.87
Adjusted^b^ HR (95% CI)	1	0.55 (0.19–1.56)	0.48 (0.18–1.32)	1.11 (0.50–2.45)	0.79
Death due to infection					
Crude HR (95% CI)	1	1.09 (0.62–1.91)	0.93 (0.52–1.65)	0.95 (0.49–1.85)	0.89
Adjusted^a^ HR (95% CI)	1	1.09 (0.62–1.91)	0.94 (0.53–1.67)	1.02 (0.54–1.92)	0.94
Adjusted^b^ HR (95% CI)	1	1.05 (0.61–1.81)	0.89 (0.51–1.54)	0.98 (0.51–1.88)	0.89

^a^Adjusted HR: adjustments were made for age, sex, time on hemodialysis, use of diuretics, and use of atorvastatin.

^b^Adjusted HR: adjustments were made for age, sex, time on hemodialysis, use of diuretics, C-reactive protein, body mass index, hemoglobin, albumin, previous coronary artery disease, and use of atorvastatin.

^c^Combined cardiovascular events were defined as a composite of death from cardiac causes, fatal or nonfatal stroke, and nonfatal myocardial infarction, whichever occurred first. Death from cardiac causes comprised death due to congestive heart failure, sudden cardiac death, fatal myocardial infarction, death due to coronary artery disease during or within 28 days after an intervention, and all other deaths attributable to coronary artery disease.

There also was no association between higher IL-16 levels and an increased risk for the separate outcome measures such as sudden cardiac death, death due to heart failure, myocardial infarction (fatal and nonfatal), or stroke (fatal and nonfatal). These results remained unchanged when adjusting for several confounders including age, sex, time on hemodialysis, use of diuretics (as a marker for residual kidney function), C-reactive protein, body mass index, hemoglobin, albumin, previous coronary artery disease, and use of atorvastatin (Table 4).

## Discussion

The present work demonstrates that IL-16 plasma levels are consistently elevated in patients on dialysis and correlate with pOx concentrations in three different cohorts. We previously found that elevated serum oxalate is associated with increased risk for cardiovascular events and sudden cardiac death in patients on dialysis^14^. However, no effect of elevated IL-16 on survival or cardiovascular events could be detected in the present study.

While elevated IL-16 concentrations have been described in psychiatric^18^, autoimmune^19–21^, metabolic^22^, and oncologic diseases^23–25^, to our knowledge, the present study is the first to describe elevated IL-16 concentrations in humans with kidney disease. IL-16 concentrations in dialysis patients are increased at least threefold compared to the healthy population. Although our cohort of healthy individuals (supplementary cohort) is rather small and younger as compared to the patient group, the mean IL-16 level of 150 pg/mL (SD 27) aligns with reported concentrations in other cohorts of healthy individuals, e.g., 187 pg/mL^21^, 101 pg/mL^23^ and 88 pg/mL^24^. Therefore, we consider our conclusion to be robust.

IL-16, which has so far received little attention, has generally been described as a danger signal^26^. It is known that IL-16 is produced by various immune cells^27^, cleaved by caspase-3^28^ and liberated in a cell-death associated process^29–31^. Extracellularly, IL-16 is described to aggregate and form a homo-tetramer^27,30^, acting on the CD4 receptor and expresses an immunomodulating and chemoattractant effect on cells^32^.

Regarding the role of IL-16 in atherosclerosis and cardiovascular disease, in vitro studies demonstrated a chemoattractant effect of IL-16 on Tregs, as well as an ability to induce Tregs^32^, which may suggest that IL-16—through the secretion of IL-10—may reduce inflammation. Moreover, some researchers also suggest "protective" attributes of IL-16, as higher circulating concentrations have been associated with a reduced incidence of post-operative cardiovascular events^33^. In contrast, IL-16 has also been implicated in the migration of vascular smooth muscle cells, leading to vascular remodeling and lesion formation^34^, which is a primary cause of vascular diseases. Furthermore, elevated IL-16 concentrations have been observed in patients with acute myocardial infarction^35^ and have been associated with cardiac fibrosis and myocardial stiffening^36^. To this end, the biological role, and clinical implications of IL-16 in the development of atherosclerosis and cardiovascular disease require further investigation.

Therefore, to investigate the role of IL-16 in cardiovascular disease, we performed a post-hoc analysis of the 4D Study and examined the impact of elevated IL-16 concentrations on survival and cardiovascular outcomes. We were unable to detect any effect of IL-16 concentration on all-cause mortality, combined cardiovascular events or its separate outcome measures such as sudden cardiac death, death due to heart failure, myocardial infarction, or stroke. Furthermore, we observed no significant effect of IL-16 on mortality related to infection. Results did not differ when adjusting for several confounders, including the main risk factors of mortality in patients receiving maintenance dialysis^37^, as well as atorvastatin, to address the original interventional study design. In a previous study, we demonstrated a link between oxalate and mortality in the 4D study^14^. Our current findings examining the same patient cohort suggested a correlation between oxalate and IL-16, leading us to explore the possibility of an association between IL-16 and mortality. Several potential explanations could account for the absence of this association: IL-16 might act as a mediator, explaining only a portion of oxalate's effect through covariance, or as a confounder—a variable linked to oxalate through non-causal pathways. Since we were unable to establish any influence of IL-16 on mortality and cardiovascular events in CKD patients in either cohort 1 or cohort 2 (4D study), further exploration in additional cohorts is warranted. Several important future endpoints could be worth investigating. First, given that studies have reported IL-16 to be overexpressed in various cancers^23,25^ and demonstrated the effectiveness of IL-16 neutralization in improving anti-tumor therapy^38^, coupled with the high prevalence of oncologic diseases among chronic kidney disease patients^39^, it may be valuable to investigate the involvement of IL-16 in cancer progression within this patient cohort. Second, given the significant involvement of the immune system in uremia-related complications, such as infectious events^40^, the role of IL-16 in these warrants further investigation. Third, the notably elevated levels of IL-16 observed in patients with systemic lupus erythematosus (SLE)^41^, along with studies of elevated levels in the urine of patients with SLE^42,43^, suggest a need for further investigation into its potential role in the progression of lupus nephritis, presenting a compelling subgroup for investigating the role of IL-16 in chronic kidney disease progression.

In cohort 2 (4D Study), IL-16 levels were measured in samples that had been stored for almost 20 years at − 80 °C. According to the literature, the measurement of IL-16 concentrations has been shown to provide stable results after prolonged, post-centrifugation storage^44^. The reliability of oxalate measurements in the 4D samples has been discussed previously^14^: due to pre-analytical conditions of the samples, oxalate concentrations tend to be higher, but it can be assumed that the relative rank order remains intact.

Different mechanisms may explain the increases of IL-16 concentrations in dialysis patients and its association with pOx. First, as GFR declines IL-16 could potentially accumulate, similar to the observation that pOx is elevated in patients with reduced GFR. In this case, the association between oxalate and IL-16 would be a mere statistical coincidence and IL-16 could be considered as a surrogate marker of GFR. The correlation between IL-16 concentration and parameters such as dialysis vintage and the absence of residual kidney function (in cohort 1 and 2) favours this consideration. Second, an increase of IL-16 could be triggered by the release from inflammatory cells in uremia. This would be in line with the observation that uremia might lead to increased cell death and cytokine release of peripheral immune cells^45^, suggesting a potential direct mechanism of IL-16 release induced by pOx. Of note, IL-16 levels do not differ between patients on HD or PD, so exogenous triggers such as dialysis membranes and filters are unlikely to explain the elevated IL-16 concentrations. Overall, it is necessary to further evaluate whether the observed association between pOx and IL-16 is more likely to be direct or indirect.

Although oxalate crystals have been shown to lead to secretion of IL-1α and IL-1β from monocytes and dendritic cells^16,17,46,47^, our findings did not show an association between pOx concentration and cytokines of the IL-1 family, including IL-33. Confirming the increase of IL-16 concentrations in three different cohorts of dialysis patients using two different methods of IL-16 measurement represents a critical strength of the present work. The 4D study we used to determine the clinical impact of elevated IL-16 levels is a large and well-established cohort of dialysis patients.

Several limitations should be considered when interpreting our findings. We measured pOx concentration but not calcium oxalate crystal formation. It is important to highlight that the described pOx concentrations are high enough to reach calcium oxalate supersaturation, potentially leading to microcrystal formation and tissue deposition^9,10^. Moreover, we are unable to prove a causal relationship between elevated IL-16 levels and pOx concentrations. IL-16 did not only statistically correlate with pOx concentrations, but also with time on dialysis and residual kidney function (Table 2). Thus, pOx and IL-16 share similar clinical characteristics, which may explain the observed association. Cohorts 1 and the supplementary cohort received a treatment as usual, which potentially included medications (such as statins) that could influence cytokine release. Our study in the 4D cohort was a post hoc analysis of an interventional trial which only included diabetic patients. Diabetes itself is considered to have a strong effect on the immune system and inflammatory processes^48^. Additionally, the role of IL-16 in diabetes is not fully understood. Patients with diabetes were found to have higher levels of IL-16^49^, and a gene polymorphism of the IL-16 gene was associated with higher risk of disease^50^. Hence, it could be worthwhile to repeat the analysis in a more heterogeneous patient cohort.

In conclusion, the present work introduces IL-16 as a novel player within the altered cytokine network in patients on dialysis. Its levels correlate with plasma oxalate, which is a risk factor for cardiovascular events and sudden cardiac death in patients on dialysis. However, no association could be detected between elevated IL-16 levels and the risk of mortality or cardiovascular events. Further studies are warranted to explain the increased IL-16 concentrations in patients with kidney disease and its clinical implications.

## Methods

### Study populations

#### Cohort 1

Patient data were obtained from a cross-sectional study in the US, as described elsewhere^14^. 106 patients aged ≥ 18 years, were enrolled from four outpatient dialysis centres located in Connecticut, USA, between April and September 2016. All participants provided written informed consent and received either thrice weekly HD for three to five hours per treatment session or daily home PD. Patients had to be medically stable with no infections or hospitalizations for a minimum of three months. Patients with a diagnosis of primary or secondary hyperoxaluria were excluded from the study. In the HD group, blood samples were collected prior to initiation of dialysis at the first appointment after a long interval. In the PD group, blood samples were collected at the monthly clinic appointment. To evaluate the validity of one-time sampling, we collected repeat blood samples from 20 patients at 3–4 subsequent treatment sessions, weekly after the long interval in HD patients and monthly at clinical appointments in PD patients. For patients with repeated measurements the first measurement was used for the analysis. The study was approved by the local authorities (Western Institutional Review Board Study No. 1162867). Data was extracted from the Yale-New Haven Hospital’s information system Epic (Epic; Verona, USA) and collected using SPSS (IBM, New York, USA). BMI was calculated by dividing a person's weight in kilograms by the square of their height in meters. All types of diabetes were included in this study as indicated by each patient's individual file.

### Cohort 2 (4D cohort)

Design and methods of the 4D Study have been reported several times before^14,51^. In brief, the 4D Study was a prospective randomized controlled trial including 1255 HD patients with type 2 diabetes mellitus, who were 18–80 years old and had begun renal replacement therapy within the previous 2 years. Between March 1998 and October 2002, patients were recruited in 178 dialysis centers in Europe and randomly assigned to double-blind treatment with 20 mg atorvastatin (n = 619) or placebo (n = 636) once daily. The study was approved by the Universities of Würzburg and Heidelberg and by all Review Boards responsible for participating centres in the study^52^.

### Statement on experiments with human participants

The studies with humans were approved by appropriate committees/local authorities mentioned above (Western Institutional Review Board, Ethics Committee of Charité, University of Würzburg, Heidelberg and responsible Review Boards for study centres of the 4D study). All patients/participants of the three cohorts gave their written informed consent before inclusion. The research was performed in accordance with all relevant guidelines and regulations following the Declaration of Helsinki.

### Data collection

In cohort 1, clinical and supplementary laboratory, data were collected from electronic health records and complemented by reports from the treating clinician. Follow-up was thrice weekly for HD and once a month for PD patients, according to standard clinical routines.

In cohort 2 (4D), demographic and clinical information was obtained through patient interviews and reports from the treating nephrologists. Coronary artery disease was defined by a history of myocardial infarction, coronary artery bypass grafting surgery, percutaneous coronary intervention, or the presence of typical vascular findings by coronary angiography^51^.

### Outcome assessment (4D Study)

In the 4D Study, the primary endpoint was a composite of cardiac death, nonfatal myocardial infarction, and fatal or nonfatal stroke, whichever occurred first (composite cardiovascular endpoint). Death from cardiac causes comprised death due to congestive heart failure, sudden cardiac death, fatal myocardial infarction, death due to coronary disease during or within 28 days after an intervention, and all other deaths that might be attributed to coronary artery disease. Sudden cardiac death was defined as: death verified by terminal rhythm disorders in an electrocardiogram, death observed by witnesses within one hour after the onset of cardiac symptoms, sudden cardiac death confirmed by autopsy, or unexpected death presumably or possibly of cardiac origin and in absence of a potassium level ≥ 7.5 mmol/L before the start of the three most recent HD sessions. Myocardial infarction was diagnosed when two of the following three criteria were met: typical symptoms, increased levels of cardiac enzymes (i.e., a level of creatine kinase MB > 5% of the total level of creatine kinase, a level of lactic dehydrogenase 1.5 times the upper limit of normal, or a level of troponin T > 2 ng/mL), or characteristic changes on the electrocardiogram. Stroke was defined as a neurologic deficit lasting > 24 h. Computed tomographic or magnetic resonance imaging was available in all but 16 cases^51^.

### Oxalate measurement

Plasma oxalate (pOx) concentrations were measured as described previously^53^. In brief, blood samples of the patients and healthy participants were immediately put on wet ice and further processed: after centrifugation, the supernatant was filtered through a Vivaspin® 500 30,999 MWCO PES filter (Sartorius, Göttingen, Germany), acidified, and measured enzymatically with oxalate oxidase (Trinity Biontech, Bray, Co. Wicklow, Ireland). The lower limit of detection of our assay was 2 µM.

In the 4D Study, oxalate concentrations were measured in baseline serum samples taken 1 week prior to randomization, and stored at − 80 °C. Frozen serum samples were slowly thawed, vigorously vortexed, and then processed as described above.

### MSD Multiplex assays

In cohort 1, 21 cytokines including granulocyte–macrophage colony-stimulating factor (GM-CSF), interferon gamma (IFN-γ), IL-1α, IL-1β, IL-2, IL-4, IL-5, IL-6, IL-7, IL-8, IL-10, IL-12p40, IL-12p70, IL-13, IL-15, IL-16, IL-17, TNF-α, TNF-β and vascular endothelial growth factor (VEGF), were measured using a V-PLEX Proinflammatory Panel 1 Human and a V-PLEX Cytokine Panel 1 Human (both Meso Scale Diagnostics, Rockville, USA); and IL-33 using a Human IL-33 Quantikine enzyme-linked immunosorbent assay (ELISA, R&D Systems, Minneapolis, USA) following the company’s instruction. In brief, the samples were diluted twofold with Diluent 2 (V-PLEX Proinflammatory Panel 1 Human) or Diluent 43 (V-PLEX Cytokine Panel 1 Human) in the plate, the samples, calibrators, or controls were applied, and the plate incubated for 2 h at room temperature (RT) while shaking. Subsequently, the plate was washed, and pooled detection antibody was added, and the plates again incubated for 2 h on the shaker. After washing, 2X Read Buffer T was added to the plate and read on an MESO QuickPlex SQ 120 reader (Meso Scale Diagnostics). The analysis was performed using the MSD DISCOVERY WORKBENCH analysis software (Meso Scale Diagnostics).

In cohort 2 (4D Study), IL-16 was also measured using the V-PLEX Cytokine Panel 1 Human. Samples were diluted 1:5 with diluent 43. Instead of the pooled antibody mixture, only the IL-16 antibody was used. The protocol was performed as described before.

### IL-33 ELISA

The IL-33 Quantikine ELISA was applied in cohort 1, and performed as per the manufacturer’s instructions. Samples were diluted twofold. A SpectraMax M3 microplate reader (Molecular Devices, San Jose, USA) was used to determine the optical density (OD) at 450 nm wavelength.

### Statistical analysis

#### Cohort 1

The statistical analysis was performed using RStudio Version 1.2.5001 (Posit Software PBC, Boston, USA). *P* values of < 0.01 were considered statistically significant. For continuous variables, the median and IQR or mean and standard deviation (SD) were calculated. For categorical variables, frequency tables were calculated and the number (n) and the relative proportion (%) reported. Cohort 1: As not all analytes were normally distributed, the Spearman correlation rank test was used to analyse the association of pOx with the 21 cytokines. In addition, two linear regression models were calculated. For model 1, a univariate linear regression was calculated to test the association between pOx (independent variable) and the cytokines (dependent variables). Second, we fitted a model adjusting for the main confounding variables, i.e., age, gender, BMI, and diabetes (model 2). To identify clinical or laboratory characteristics associated with IL-16, the study group was stratified based on IL-16 concentrations into three subgroups. We compared the characteristics between those subgroups by analyses of variation (ANOVA, for continuous outcomes) or Pearson chi square statistic (for categorical outcomes). Differences between the groups were further assessed by testing the association of the clinical or laboratory parameters (independent variable) and IL-16 (dependent variable).

### Cohort 2 (4D Study)

We calculated means (SDs) or medians (interquartile ranges [IQRs]) for continuous and frequency tables for categorical variables. We compared characteristics between groups by analyses of variation (ANOVA), or chi-square tests where appropriate. Patient characteristics are presented in subgroups defined by quartiles of IL-16 concentrations at baseline, with the following cut-points: ≤ 326 pg/mL, > 326 to ≤ 416 pg/mL, > 416 to ≤ 535 pg/mL, and > 535 pg/mL Bivariate correlations of IL-16, pOx and uremic toxins (creatinine, ADMA, SDMA, serum carbamylated albumin (C-Alb) und BUN/Urea) were analysed by calculating scatter plots and Pearson correlation coefficients. A multivariate prediction model was calculated to assess the impact of uremic toxins on IL-16. The risk of all-cause mortality, reaching the composite cardiovascular endpoint, death due to congestive heart failure, sudden cardiac death, myocardial infarction, stroke, and death due to infection, according to quartiles of IL16 levels (with quartile 1 being the reference group) were assessed by Cox regression models. First, we fitted a model including the main confounding variables i.e., age, sex, and two markers indicating progressive uremia, i.e. time on HD, and use of diuretics (model 1). Second, we fitted a model additionally adjusting for C-reactive protein, BMI, hemoglobin, albumin, and previous coronary artery disease (model 2—core model), which are known to be established risk factors for mortality in dialysis patients. All models were adjusted for treatment group (atorvastatin vs. placebo) and fit as complete case analyses (no missing value imputation)^14,37^. *P*-values are two-sided.

Statistical analyses were conducted using STATA (StataCorp. 2017. Stata Statistical Software: Release 15. College Station, TX: StataCorp LLC) for the 4D Study.

### Supplementary Information

Supplementary Information.

## Data Availability

The data underlying this article will be shared upon reasonable request to the corresponding author.

## References

[1] Himmelfarb J, Vanholder R, Mehrotra R, Tonelli M (2020). The current and future landscape of dialysis. Nat. Rev. Nephrol..

[2] Johansen KL (2022). US renal data system 2021 annual data report: Epidemiology of kidney disease in the United States. Am. J. Kidney Dis. Off. J. Natl. Kidney Found..

[3] Cobo G, Lindholm B, Stenvinkel P (2018). Chronic inflammation in end-stage renal disease and dialysis. Nephrol. Dial Transplant..

[4] Zimmermann J, Herrlinger S, Pruy A, Metzger T, Wanner C (1999). Inflammation enhances cardiovascular risk and mortality in hemodialysis patients. Kidney Int..

[5] Duranton F (2012). Normal and pathologic concentrations of uremic toxins. J. Am. Soc. Nephrol..

[6] Pertosa G, Grandaliano G, Gesualdo L, Schena FP (2000). Clinical relevance of cytokine production in hemodialysis. Kidney Int..

[7] Stenvinkel P (2005). IL-10, IL-6, and TNF-α: Central factors in the altered cytokine network of uremia–the good, the bad, and the ugly. Kidney Int..

[8] Perinpam M (2017). Plasma oxalate in relation to eGFR in patients with primary hyperoxaluria, enteric hyperoxaluria and urinary stone disease. Clin. Biochem..

[9] Hoppe B (1999). Plasma calcium oxalate supersaturation in children with primary hyperoxaluria and end-stage renal failure. Kidney Int..

[10] Worcester EM, Nakagawa Y, Bushinsky DA, Coe FL (1986). Evidence that serum calcium oxalate supersaturation is a consequence of oxalate retention in patients with chronic renal failure. J. Clin. Invest..

[11] Waikar SS (2019). Association of urinary oxalate excretion with the risk of chronic kidney disease progression. JAMA Intern. Med..

[12] Liu Y (2021). Dysregulated oxalate metabolism is a driver and therapeutic target in atherosclerosis. Cell Rep..

[13] Mulay SR (2016). Oxalate-induced chronic kidney disease with its uremic and cardiovascular complications in C57BL/6 mice. Am. J. Physiol.-Renal. Physiol..

[14] Pfau A (2021). High oxalate concentrations correlate with increased risk for sudden cardiac death in dialysis patients. J. Am. Soc. Nephrol..

[15] Ludwig-Portugall I (2016). An NLRP3-specific inflammasome inhibitor attenuates crystal-induced kidney fibrosis in mice. Kidney Int..

[16] Mulay SR (2013). Calcium oxalate crystals induce renal inflammation by NLRP3-mediated IL-1β secretion. J. Clin. Invest..

[17] Schunk SJ (2021). Interleukin-1α is a central regulator of leukocyte-endothelial adhesion in myocardial infarction and in chronic kidney disease. Circulation.

[18] Stelzhammer V (2014). Proteomic changes in serum of first onset, antidepressant drug-naïve major depression patients. Int. J. Neuropsychopharm..

[19] Kawabata K (2020). IL-16 expression is increased in the skin and sera of patients with systemic sclerosis. Rheumatology.

[20] Lee S (1998). Circulating interleukin-16 in systemic lupus erythematosus. Rheumatology.

[21] Purzycka-Bohdan D (2016). Assessment of interleukin 16 serum levels and skin expression in psoriasis patients in correlation with Clinical severity of the disease. PLoS ONE.

[22] Lichtenauer M (2015). Elevated plasma levels of interleukin-12p40 and interleukin-16 in overweight adolescents. Biomed. Res. Int..

[23] Alexandrakis MG (2004). Serum level of interleukin-16 in multiple myeloma patients and its relationship to disease activity. Am. J. Hematol..

[24] Long S-F, Chen G-A, Fang M-S (2015). Levels of interleukin-16 in peripheral blood of 52 patients with multiple myeloma and its clinical significance. Int. J. Clin. Exp. Med..

[25] Yang H, Han Y, Wu L, Wu C (2017). Diagnostic and prognostic value of serum interleukin-16 in patients with gastric cancer. Mol. Med. Rep..

[26] Roth S, Solbach W, Laskay T (2016). IL-16 and MIF: Messengers beyond neutrophil cell death. Cell Death Dis..

[27] Cruikshank WW, Kornfeld H, Center DM (2000). Interleukin-16. J. Leukoct. Biol,.

[28] Zhang Y (1998). Processing and activation of pro-interleukin-16 by caspase-3. J. Biol. Chem..

[29] Elssner A, Doseff AI, Duncan M, Kotur M, Wewers MD (2004). IL-16 is constitutively present in peripheral blood monocytes and spontaneously released during apoptosis. J. Immunol..

[30] Richmond J, Tuzova M, Cruikshank W, Center D (2014). Regulation of cellular processes by interleukin-16 in homeostasis and cancer. J. Cell. Physiol..

[31] Roth S (2015). Secondary necrotic neutrophils release interleukin-16C and macrophage migration inhibitory factor from stores in the cytosol. Cell Death Discov..

[32] McFadden C (2007). Preferential migration of T regulatory cells induced by IL-16. J. Immunol..

[33] Grönberg C (2016). Endarterectomy patients with elevated levels of circulating IL-16 have fewer cardiovascular events during follow-up. Cytokine.

[34] Park SL (2015). p21WAF1 is required for interleukin-16-induced migration and invasion of vascular smooth muscle cells via the p38MAPK/Sp-1/MMP-9 pathway. PLoS ONE.

[35] Schernthaner C (2017). Elevated plasma levels of interleukin-16 in patients with acute myocardial infarction. Medicine.

[36] Tamaki S (2013). Interleukin-16 promotes cardiac fibrosis and myocardial stiffening in heart failure with preserved ejection fraction. PLOS ONE.

[37] Ma L, Zhao S (2017). Risk factors for mortality in patients undergoing hemodialysis: A systematic review and meta-analysis. Int. J. Cardiol..

[38] Yang S-J (2024). Neutralizing IL-16 enhances the efficacy of targeting Aurora-A therapy in colorectal cancer with high lymphocyte infiltration through restoring anti-tumor immunity. Cell Death Dis..

[39] Stengel B (2010). Chronic kidney disease and cancer: a troubling connection. J. Nephrol..

[40] Syed-Ahmed M, Narayanan M (2019). Immune dysfunction and risk of infection in chronic kidney disease. Adv. Chronic Kidney Dis..

[41] Lard LR, Roep BO, Verburgh CA, Zwinderman AH, Huizinga TWJ (2002). Elevated IL-16 levels in patients with systemic lupus erythematosus are associated with disease severity but not with genetic susceptibility to lupus. Lupus.

[42] Fava A (2024). Urine proteomic signatures of histological class, activity, chronicity, and treatment response in lupus nephritis. JCI Insight.

[43] Fava A (2022). Urine proteomics and renal single cell transcriptomics implicate IL-16 in lupus nephritis. Arthr. Rheumatol..

[44] Kofanova O (2018). IL8 and IL16 levels indicate serum and plasma quality. Clin. Chem. Lab. Med. (CCLM).

[45] Betjes MGH, Langerak AW, van der Spek A, de Wit EA, Litjens NHR (2011). Premature aging of circulating T cells in patients with end-stage renal disease. Kidney Int..

[46] Knauf F (2013). NALP3-mediated inflammation is a principal cause of progressive renal failure in oxalate nephropathy. Kidney Int..

[47] Luz HL (2019). P2X7 receptor stimulation is not required for oxalate crystal-induced kidney injury. Sci. Rep..

[48] Berbudi A, Rahmadika N, Tjahjadi AI, Ruslami R (2020). Type 2 diabetes and its impact on the immune system. Curr. Diabetes Rev..

[49] Zak, K. P., Kondratskaia, I. N., Mel’nichenko, S. V. & Popova, V. V. [Circulating interleukin-16 in blood of patients with metabolic syndrome and type 2 diabetes mellitus]. *Lik Sprava* 46–49 (2007). 18416164

[50] Mohammad DG, Omar H, El-Abaseri TB, Omar W, Abdelraheem S (2021). Association of IL-16 rs11556218 T/G polymorphism with the risk of developing type 2 diabetes mellitus. J. Diabetes Metab. Disord..

[51] Wanner C (2005). Atorvastatin in patients with type 2 diabetes mellitus undergoing hemodialysis. N. Eng. J. Med..

[52] Wanner C (1999). Rationale and design of a trial improving outcome of type 2 diabetics on hemodialysis. Kidney Int..

[53] Pfau A (2020). Assessment of plasma oxalate concentration in patients With CKD. Kidney Int. Rep..

