# Supplementary material for: Interleukin-16 is increased in dialysis patients but is not a cardiovascular risk factor
Source: Sci Rep. 2024 May 17;14:11323. doi: 10.1038/s41598-024-61808-7 (PMC11101424; doi:10.1038/s41598-024-61808-7)
Supplement: Supplementary file 1 — Supplementary Information. [file 41598_2024_61808_MOESM1_ESM.docx]

**SUPPLEMENTAL MATERIAL**

**Interleukin-16 is increased in dialysis patients but is not a cardiovascular risk factor.**

Frederic Brösecke^1^; Anja Pfau^1,2^; Theresa Ermer^3,4^; Ana Beatriz Dein Terra Mota Ribeiro^1^; Lisa Rubenbauer^1^; Veena S. Rao^5^, Sarah Burlein^1^; Bernd Genser^6,7^, Martin Reichel^1^; Peter S. Aronson^4^; Steven Coca^8^; Felix Knauf*^1,4^

^1^ Charité – Universitätsmedizin Berlin, corporate member of Freie Universität Berlin and Humboldt-Universität zu Berlin, Department of Nephrology and Medical Intensive Care, Berlin, Germany

^2^ MVZ Dialysezentrum (Dialysis Center) Schweinfurt, Schweinfurt, Germany

^3^ Department of Nephrology and Hypertension, Friedrich-Alexander-Universität Erlangen-Nürnberg, Erlangen, Germany

^4^ Department of Internal Medicine, Section of Nephrology, Yale University School of Medicine, New Haven, CT, USA

^5^ Department of Internal Medicine, Section of Cardiovascular Medicine, Yale University School of Medicine, New Haven, CT, USA

^6^ Department of General Medicine, Centre for Preventive Medicine & Digital Health Baden Württemberg, Ruprecht Karls University Heidelberg, Germany

^7^ High5Data GmbH, Heidelberg, Germany

^9^ Mt. Sinai Hospital, Mount Sinai School of Medicine, New York, New York, USA

**Table of content:**

- Supplementary cohort: Study population, data collection, IL-16-measurement, statistical analysis.
- Supplementary Table 1: Intraindividual variability of cytokines and oxalate between dialysis sessions.
- Supplementary Table 2: Characteristics of 12 patients on hemodialysis and 6 healthy control subjects in Germany (supplementary cohort)
- Supplementary Figure 1: Association of IL-16 with clinical parameters.
- Supplementary Figure 2: IL-16 concentrations and the risk of mortality in 104 US patients (cohort 1).
- Supplementary Figure 3: The increase of Interleukin-16 concentration and its correlation with plasma oxalate is reproducible in a small cohort of dialysis patients (supplementary cohort).

**Supplementary cohort**

**Study population**

The study population included patients with kidney failure on maintenance HD at a university dialysis centre (Charité, Berlin, Germany), who were enrolled between February and March 2018. Included were patients that provided written informed consent, were ≥18 years old, were on thrice weekly HD treatment for at least three months, were medically stable and not hospitalized in the preceding four weeks. Patients with an underlying hyperoxaluric condition were excluded. Blood samples were collected before the dialysis session after the long dialysis interval. Healthy volunteers were included as controls. The study protocol was approved by the local authorities (local Ethics Committee of Charité, Berlin Study No. EA2/242/17).

**Data collection**

In the supplementary cohort, clinical and supplementary laboratory data were extracted from SAP logon 740, the general clinical documentation system at Charité (SAP; Walldorf, Germany).

**IL-16 Measurement**

A Human IL-16 Quantikine ELISA Kit was used for the measurement of the plasma samples in cohort 3. The assay was performed according to the company’s protocol. The samples were diluted in a 1:2 ratio with Assay Diluent RD1W. The standard was prepared according to the protocol and the samples pipetted on the plate. The plate was incubated for 2h at RT and washed. Following this, the Human IL-16 Conjugate was added and incubated for 2h at RT. After washing, substrate solution was added, the plate again incubated for 30 min in the dark. The reaction was stopped by the addition of the stop solution. The OD was determined using a microplate reader (BioRad xMark, Hercules, USA).

**Statistical analysis**

A Spearman correlation rank test was used to analyse the association between pOx (independent variable) and IL-16 (dependent variable). Differences in concentration of pOx and IL-16 between the control and patient group were analysed using a Wilcoxon signed-rank test.

**Supplementary Table 1: Intraindividual variability of cytokines and oxalate between dialysis sessions.**

| **Analytes** | **Modality** | **Number patients** | **Median cv** | **IQR** |
| --- | --- | --- | --- | --- |
| GM-CSF | PD | 10 | 82.7 | 36 |
| GM-CSF | HD | 9 | 60.3 | 35.7 |
| IFN-γ | PD | 10 | 48.3 | 17.5 |
| IFN-γ | HD | 9 | 28.7 | 26.7 |
| IL-1α | PD | 10 | 25.6 | 35.6 |
| IL-1α | HD | 10 | 15.1 | 5.9 |
| IL-10 | PD | 10 | 31.7 | 32.1 |
| IL-10 | HD | 10 | 21.6 | 17.1 |
| IL-12p40 | PD | 10 | 11 | 5.6 |
| IL-12p40 | HD | 10 | 6.7 | 7.2 |
| IL-12p70 | PD | 4 | 55.4 | 27.2 |
| IL-12p70 | HD | 7 | 59.1 | 50.8 |
| IL-13 | PD | 2 | 40.3 | 40 |
| IL-13 | HD | 2 | 43.1 | 48.4 |
| IL-15 | PD | 10 | 6.8 | 4 |
| IL-15 | HD | 10 | 8 | 7.4 |
| IL-16 | PD | 10 | 9.7 | 14.2 |
| IL-16 | HD | 10 | 9.2 | 2.3 |
| IL-17 | PD | 10 | 45.9 | 54.2 |
| IL-17 | HD | 10 | 32.7 | 27.4 |
| IL-1β | PD | 5 | 70.7 | 13.4 |
| IL-1β | HD | 4 | 65.1 | 44.1 |
| IL-2 | PD | 10 | 64.5 | 47.5 |
| IL-2 | HD | 9 | 28.2 | 34.1 |
| IL-33 | PD | 2 | 67.7 | 12 |
| IL-33 | HD | 2 | 133 | 15.6 |
| IL-4 | PD | 7 | 71.9 | 133.2 |
| IL-4 | HD | 9 | 100 | 33.8 |
| IL-5 | PD | 10 | 30.6 | 18.2 |
| IL-5 | HD | 10 | 25 | 28.5 |
| IL-6 | PD | 10 | 36.5 | 27.7 |
| IL-6 | HD | 10 | 18.3 | 28 |
| IL-7 | PD | 10 | 25.3 | 31.1 |
| IL-7 | HD | 10 | 14.7 | 11.8 |
| IL-8 | PD | 10 | 19.3 | 11.6 |
| IL-8 | HD | 10 | 20.4 | 8.7 |
| pOx | PD | 10 | 16.1 | 25.5 |
| pOx | HD | 10 | 10.5 | 11.1 |
| TNF-α | PD | 10 | 11.6 | 4.8 |
| TNF-α | HD | 10 | 8.3 | 6.3 |
| TNF-β | PD | 10 | 31.1 | 13.7 |
| TNF-β | HD | 10 | 24.3 | 26.2 |
| VEGF | PD | 10 | 18 | 15.4 |
| VEGF | HD | 10 | 14.4 | 6.9 |

Intraindividual differences between dialysis sessions in cohort 1 was assessed in 20 patients. For those patients, the measurement of all analytes (21 cytokines and plasma oxalate (pOx)) was repeated on four dates. Patients for whom ≥3 measurements were available were considered for the evaluation. For the analysis, peritoneal dialysis (PD) and haemodialysis patients (HD) were assessed separately. The coefficients of variation (CV) were calculated for each analyte and patient by dividing the standard deviation of one’s measurement by its mean value. Results are presented as the median and the interquartile range (IQR).

**Supplementary Table 2: Characteristics of 12 patients on haemodialysis and 6 healthy control subjects in Germany (supplementary cohort)**

|  | **Patients**  **n=12** | **Healthy control individuals**  **n=6** |
| --- | --- | --- |
| IL-16 [pg/mL] | 521.5 (184) | 150.1 (27) |
| pOx [µM]* | 19.6 (31) | 1.2 (0.9) |
| Age [years] | 67.3 (15) | 40.2 (12) |
| Male, No. (%) | 7 (58) | 3 (50) |
| BMI [kg/m2] | 25.1 (4.2) | - |
| Duration of dialysis [months]* | 22.5 (62) | - |
| Urine output <300 ml/day, No. (%) | 5 (41.6) | - |
| CRP [mg/l]* | 6.5 (10) | - |

pOx: plasma oxalate; BMI: body mass index, calculated as weight in kilograms divided by height in meters squared; CRP: C-reactive protein.

Continuous variables are expressed as mean (SD) or median (IQR)* where appropriate, categorical variables as No. (%).


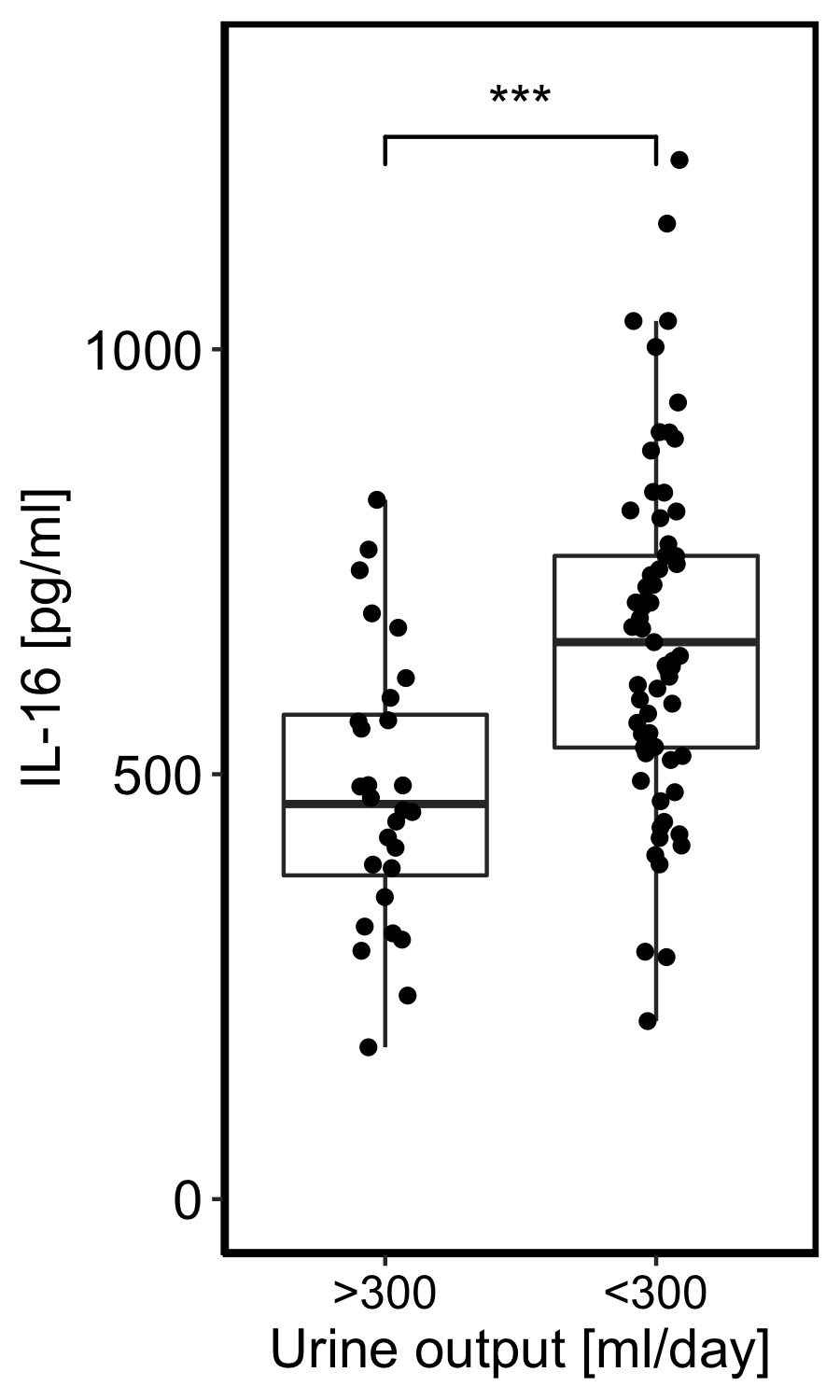

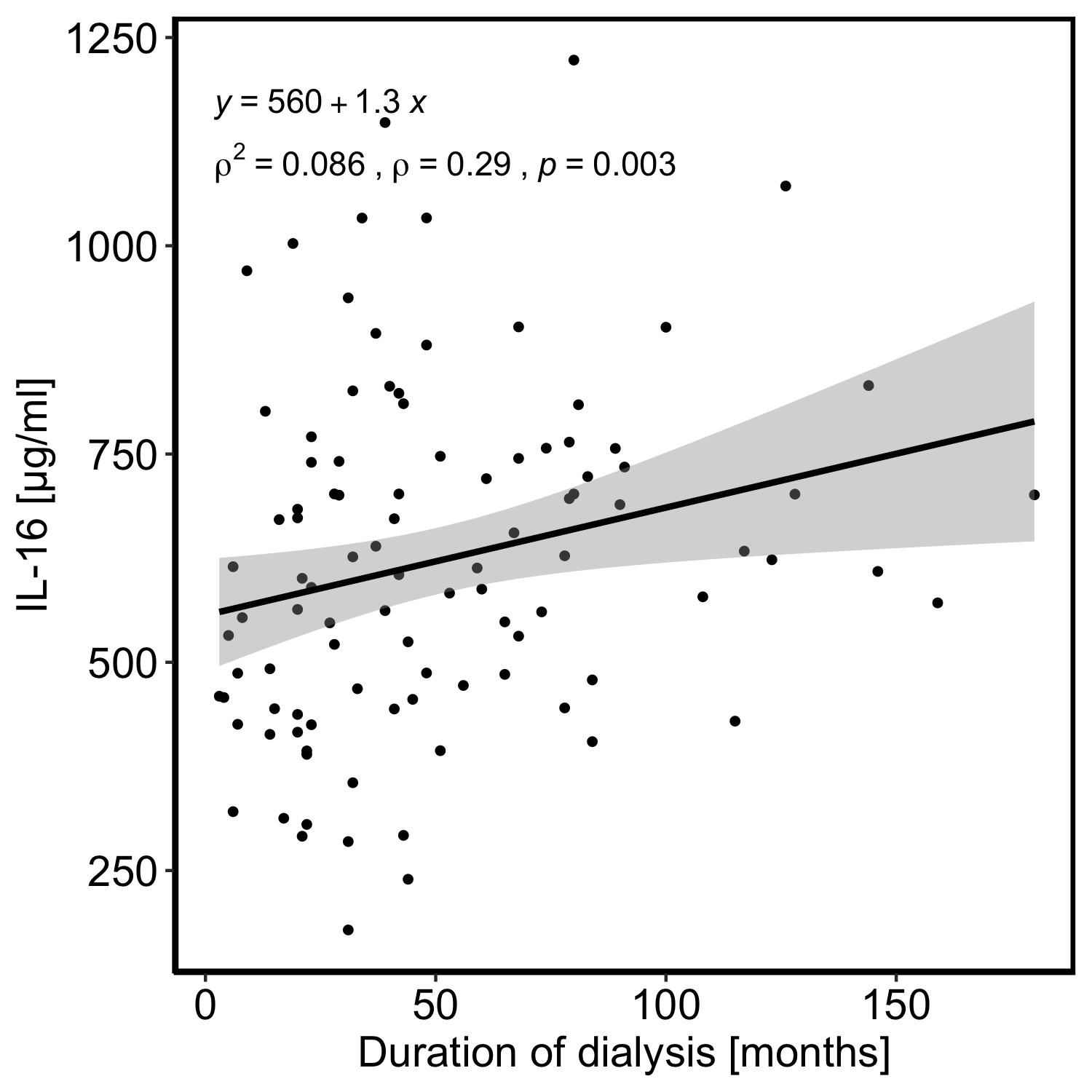


**B**

**A**

**Supplementary Figure 1: Association of IL-16 with clinical parameters.**

Cohort 1, consisting of 104 patients with kidney failure treated with long-term dialysis, was analysed for associations between interleukin 16 (IL-16) and key clinical parameters. Duration of dialysis (**A**) was found to be associated with IL-16 (Spearman`s rank test); the black line represents the regression line. Additionally, a higher concentration of IL-16 was found to be associated with lower residual kidney function (urine output <300 ml/day, **B,** Wilcoxon-Mann-Whitney test; *** <0.001).


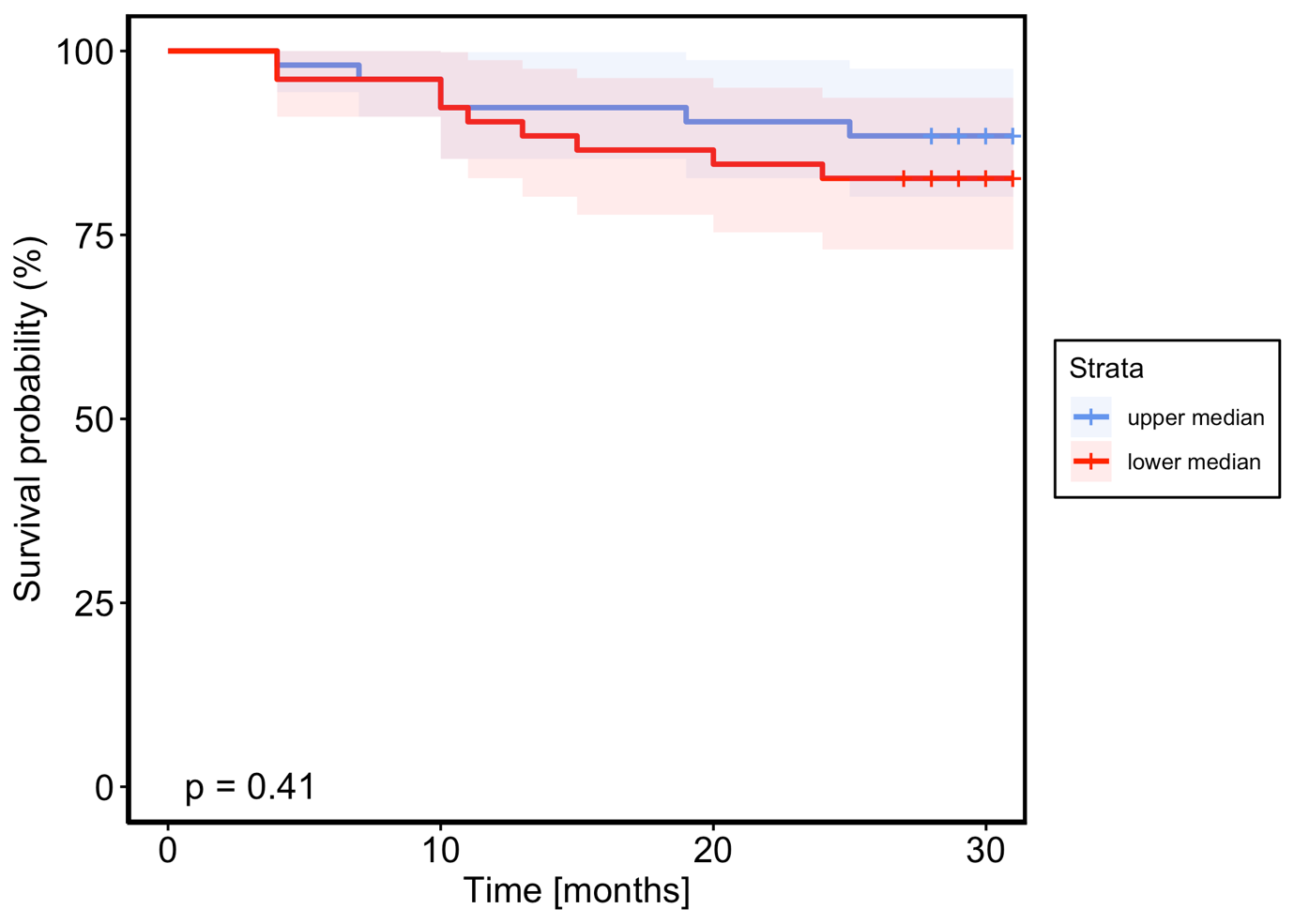


**Supplementary Figure 2: IL-16 concentrations and the risk of mortality in 104 US patients (cohort 1).**

After a 2.5-year follow-up, survival analysis was conducted, stratifying participants based on IL-16 concentration into upper and lower median groups (with a cut-off of 603 pg/ml), employing the Kaplan-Meier method. Within the cohort, 15 patients had passed away by the end of the follow-up period. Regression analysis yielded no difference in survival probability between the upper and lower median levels at baseline.

**
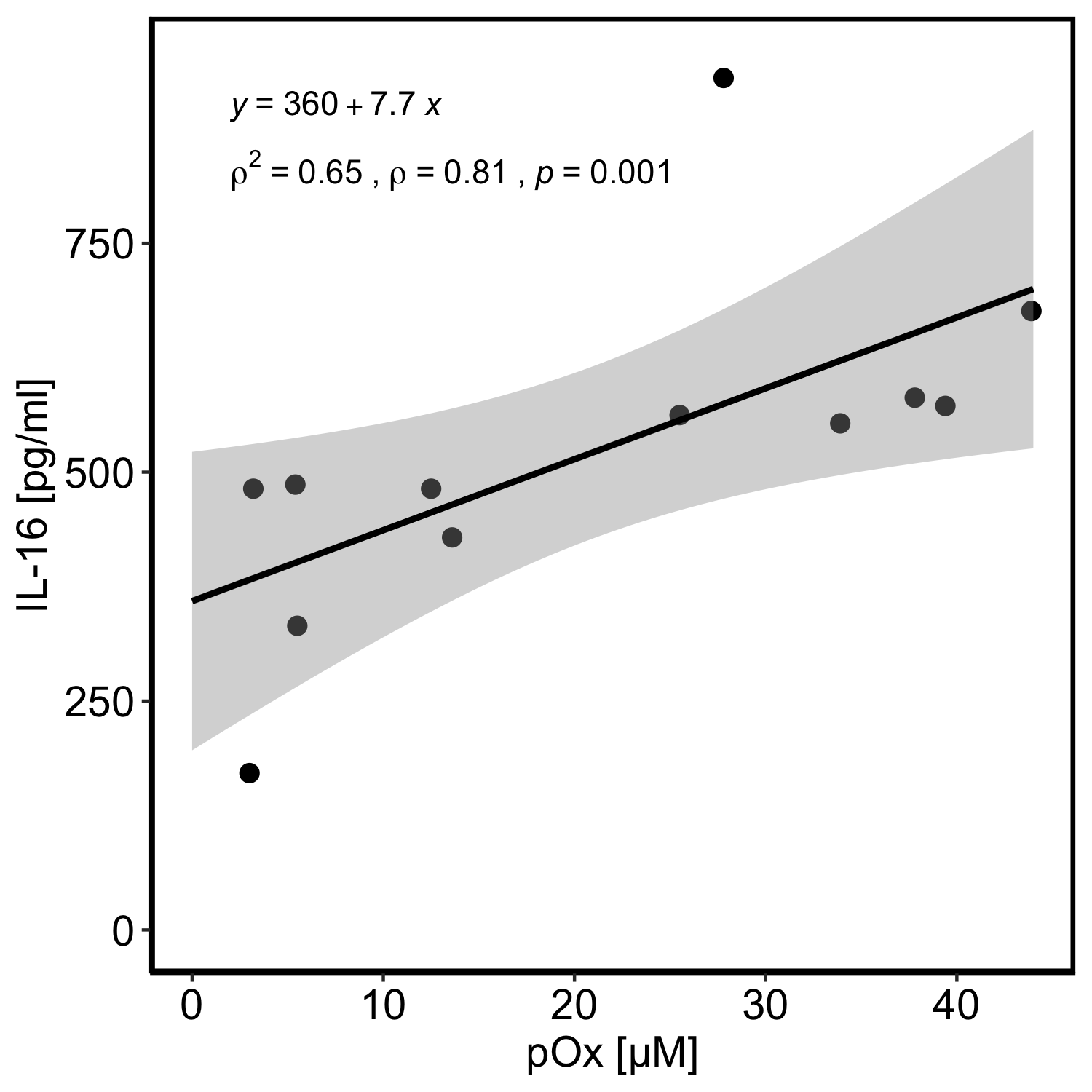

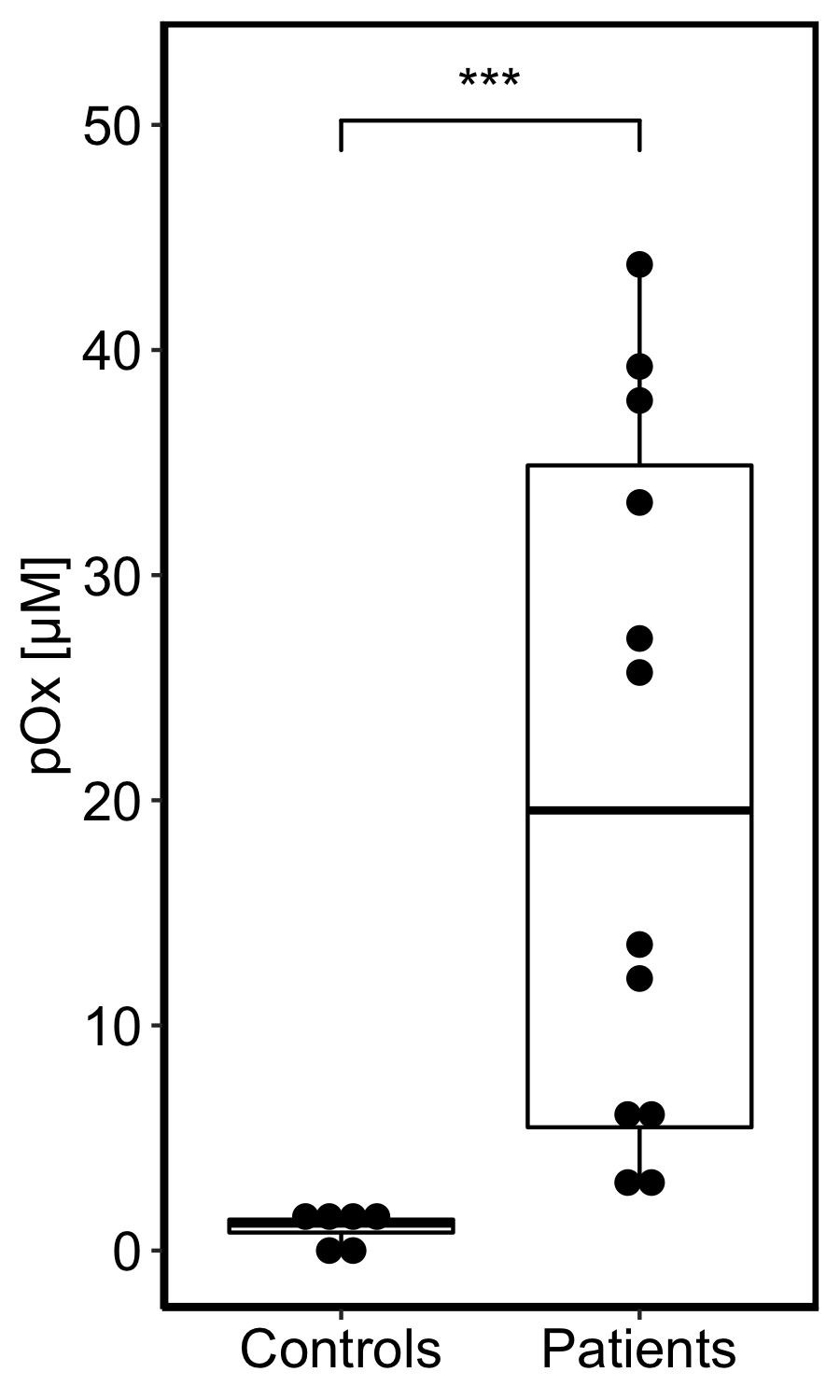

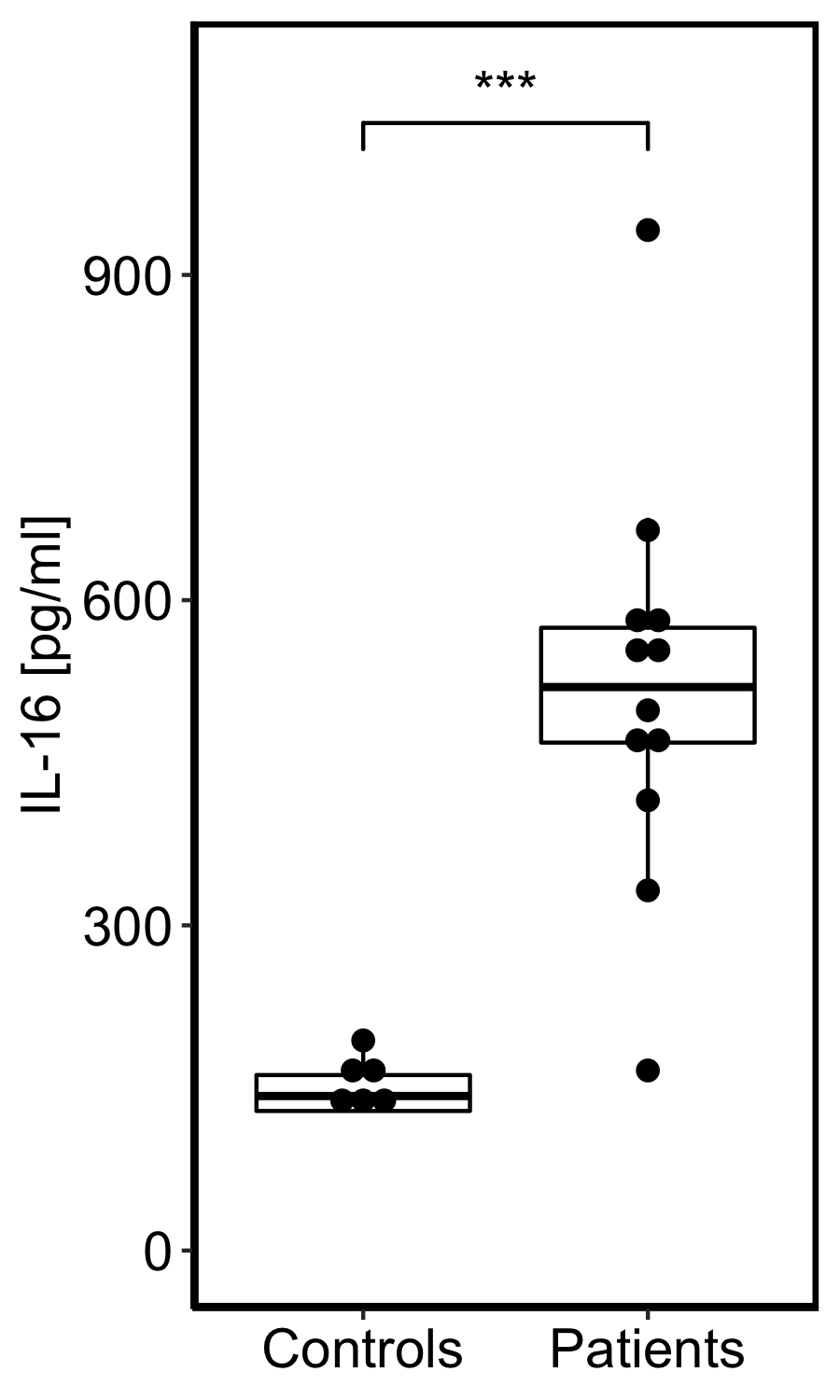
**

**B**

**A**

**Supplementary Figure 3: The increase of Interleukin-16 concentration and its correlation with plasma oxalate is reproducible in a small cohort of dialysis patients (supplementary cohort).**

**A:** In a small cohort of 12 patients with kidney failure requiring long-term dialysis in Germany (supplementary cohort), Interleukin-16 (IL-16), measured by Quantikine ELISA, is again correlated with plasma oxalate (pOx) (Spearman`s rank test, ρ=0.81). **B:** Compared to six healthy controls, pOx and IL-16 were found highly elevated in dialysis patients (Wilcoxon–Mann–Whitney test; *** <0.001).
